# Prognostic role of the neutrophil-to-lymphocyte ratio in pediatric Hodgkin lymphoma: insights from a 20-year single-center experience

**DOI:** 10.1186/s12887-026-07367-4

**Published:** 2026-07-18

**Authors:** Alper Uygun, Ayhan Dağdemir, İbrahim Kartal, Oğuz Salih Dinçer, Hülya Kangal Şimşek, Merve Ecem Öğretici Çolak, Gamzenur Yalçınkaya

**Affiliations:** Faculty of Medicine, Department of Pediatric Hematology and Oncology, Ondokuz Mayıs University, Kurupelit, Samsun, Turkey

**Keywords:** Hodgkin lymphoma, Pediatric oncology, Neutrophil-to-lymphocyte ratio

## Abstract

**Introduction:**

The neutrophil-to-lymphocyte ratio (NLR) is an established prognostic marker in adult malignancies, including Hodgkin lymphoma (HL). However, its clinical significance in pediatric HL remains uncertain due to biological differences and generally favorable outcomes. This study aimed to evaluate the prognostic value of the diagnostic NLR in predicting relapse, refractory disease, and mortality in children with HL.

**Materials and methods:**

Eighty-two patients under 18 years of age who were diagnosed with HL between 2005 and 2025 were retrospectively analyzed. Clinical features, laboratory parameters, treatment response, and survival outcomes were assessed. The NLR was calculated from baseline blood counts obtained before therapy. Receiver operating characteristic (ROC) curve analysis, Kaplan–Meier survival estimates, and logistic regression models were used to determine the prognostic performance of the NLR alongside conventional factors such as stage, B symptoms, albumin, and the erythrocyte sedimentation rate.

**Results:**

The mean NLR at diagnosis was 3.76 ± 2.77. ROC analysis revealed poor discriminative power (AUC = 0.517, *p* = 0.822). Event-free survival (EFS) and overall survival (OS) did not differ between the high and low NLR groups (*p* = 0.870 and *p* = 0.315, respectively). In the multivariable logistic regression, early two-cycle chemotherapy response remained an independent predictor of adverse outcomes (*p* = 0.002), whereas bulky disease showed borderline significance (*p* = 0.059). The NLR and other inflammatory markers, including the ESR and albumin, were not associated with poor prognosis.

**Conclusion:**

The diagnostic NLR lacks independent prognostic value in this pediatric HL cohort. These findings highlight the biological distinctness of pediatric disease and suggest that adult-derived inflammatory biomarkers should not be directly extrapolated to childhood malignancies.

## Introduction

Hodgkin lymphoma (HL) is one of the most common malignant hematological diseases in childhood and adolescence. In developed countries, its annual incidence has been reported to be 1–2 per 100,000 children [1]. Classical Hodgkin lymphoma is the most frequently observed subtype in this age group, and high cure rates can be achieved through established chemotherapy and radiotherapy protocols [1, 2]. Although treatment responses are generally favorable, clinical outcomes may vary depending on disease stage, the presence of B symptoms, and biological characteristics [1, 3]. For diagnosis and risk stratification, prognostic scoring systems incorporating parameters such as age, stage, leukocyte count, hemoglobin level, and the presence of bulky disease are commonly used [1].

Several ratios derived from complete blood count parameters have been investigated as potential biomarkers in various malignancies [4–6]. These ratios reflect the distribution of neutrophils and lymphocytes in the peripheral blood and help assess the balance between inflammatory response and immune system activity [7, 8]. Numerous studies involving adult patient cohorts have evaluated the relationships between these ratios and clinical parameters such as disease stage, survival time, and risk of relapse in patients with hematologic malignancies [3, 9, 10]. The applicability of these ratios in patients with Hodgkin lymphoma has also been explored in several studies [2, 3, 9].

The underlying prognostic value of the neutrophil-to-lymphocyte ratio (NLR) is derived from its reflection of the host’s systemic immune status and the protumorigenic microenvironment [4, 10, 11]. Elevated neutrophils are key mediators of inflammation and actively promote tumor progression through the release of proangiogenic factors and immunosuppressive cytokines [3, 4, 12]. Conversely, a decreased lymphocyte count represents a weakened functional cellular immune response necessary for eradicating malignant cells. Therefore, a high NLR signifies an unfavorable balance, which is correlated with increased disease aggressiveness and poorer clinical outcomes [10].

Inflammation-based ratios such as the NLR have been associated with prognosis in various malignancies, including Hodgkin lymphoma, particularly in adult patients [3, 8, 10]. However, the clinical relevance of these ratios in pediatric Hodgkin lymphoma patients has not yet been clearly established [11, 12]. Most of the available studies have focused on adult cohorts, with pediatric patients either excluded or analyzed only as a subgroup [3, 9, 10]. One of the largest pediatric series is the AIEOP LH-2004 cohort, in which biological markers at diagnosis were retrospectively analyzed in 769 patients [1]. In that study, the NLR (expressed as the ANC/ALC ratio) was among the evaluated variables and was significant on univariable analysis, but it was not retained as an independent prognostic factor in the final multivariable model, which comprised stage IV disease, ferritin, platelets, and eosinophils [1]. These findings underscore the need for additional pediatric-specific data on inflammatory ratios such as the NLR.

In this study, we evaluated the relationships between the NLR measured at diagnosis and clinical features, treatment response, and survival outcomes in pediatric patients with Hodgkin lymphoma. Considering that immune regulation and inflammatory responses differ between children and adults, we further aimed to determine whether the NLR maintains its prognostic significance within the distinct immunologic profile of the pediatric population.

## Materials and methods

This retrospective observational study included pediatric patients diagnosed with histopathologically confirmed HL at the Pediatric Oncology Department of Ondokuz Mayıs University Faculty of Medicine between 2005 and 2025. Patients under 18 years of age at diagnosis were eligible. The exclusion criteria included relapsed disease at presentation, prior malignancy, clinically or genetically confirmed primary immunodeficiency as determined by pediatric immunology specialists, or incomplete baseline data. Sixteen patients had incomplete or inaccessible laboratory data and were therefore not included in the statistical analyses, leaving 82 patients for evaluation.

The following variables were recorded: age at diagnosis, sex, Ann Arbor stage, presence of B symptoms, histological subtype, presence of bulky disease, chemotherapy regimens, number of cycles, early treatment response after two cycles of chemotherapy, use of radiotherapy, follow-up duration, and clinical outcomes including relapse, refractory disease, and death. Although chemotherapy protocols evolved over the study period, risk-adapted treatment principles remained consistent, and analyses were not stratified by treatment era.

Laboratory data at diagnosis included complete blood count and biochemical parameters: absolute neutrophil count, absolute lymphocyte count, white blood cell count, hemoglobin, platelet count, monocyte count, erythrocyte sedimentation rate (ESR), albumin, lactate dehydrogenase (LDH), and C-reactive protein (CRP). Complete blood counts were obtained at initial diagnosis, prior to corticosteroid, chemotherapy, or antibiotic administration, and in the absence of clinical or laboratory evidence of infection. The NLR was calculated by dividing the absolute neutrophil count by the absolute lymphocyte count (both expressed as ×10⁹/L).

Adverse clinical outcomes were defined as relapse, refractory disease, or death during follow-up. Remission was defined as the complete resolution of all clinical, radiological, and metabolic evidence of disease at the end of treatment, corresponding to a complete metabolic response on end-of-therapy PET (Deauville score ≤ 3). Refractory disease was defined as the failure to achieve remission at the end of first-line therapy despite receiving chemotherapy at doses appropriate for the patient’s risk group, whereas relapse was defined as the development of a new histopathologically confirmed lesion in a patient who had previously achieved remission. Bulky disease was defined as a mediastinal mass exceeding one-third of the thoracic diameter on chest radiography or any tumor with a maximal diameter greater than 6 cm on computed tomography. The early treatment response after two cycles of chemotherapy was evaluated via interim positron emission tomography (PET) imaging. A favorable early response was defined as an interim PET Deauville score of 1–3, thereby including Deauville score 3 among good responders, together with a ≥ 50% decrease in measurable tumor size and the absence of new lesions [13, 14].

Chemotherapy consisted of risk-adapted multiagent regimens, OPPA (vincristine, procarbazine, prednisone, doxorubicin), OEPA (vincristine, etoposide, prednisone, doxorubicin), COPP (cyclophosphamide, vincristine, procarbazine, prednisone), and ABVD (doxorubicin, bleomycin, vinblastine, dacarbazine) administered according to the institutional risk-adapted protocols in effect during the study period. Autologous stem cell transplantation was performed as salvage therapy for relapsed or refractory disease, following re-induction chemotherapy. Radiotherapy was administered as consolidation according to the risk- and response-adapted protocols in use during the study period; indications included bulky disease at diagnosis, residual disease after chemotherapy, and inadequate early metabolic response on interim PET, in line with response-adapted criteria.

Statistical analyses were performed via IBM SPSS Statistics version 22.0. Descriptive statistics are presented as the mean ± standard deviation, median (range), or n (%). Normality was assessed via the Kolmogorov–Smirnov test. Categorical variables were compared via the chi-square test, and continuous variables were compared via the Mann–Whitney U test or Student’s t test, as appropriate. The optimal NLR cutoff was determined via receiver operating characteristic (ROC) curve analysis. Survival outcomes were estimated via the Kaplan–Meier method and compared via the log-rank test. Variables with *p* < 0.10 in the univariable analysis were included in the multivariable logistic regression to identify independent predictors of adverse outcomes. To minimize overfitting, a parsimonious multivariable logistic regression model was constructed, including variables with *p* < 0.10 in the univariable analysis and clinically relevant parameters. All tests were two-tailed, and a p-value < 0.05 was considered statistically significant.

Ethical approval for the study was obtained from the Clinical Research Ethics Committee of Ondokuz Mayıs University (Decision No: 2025/341). This retrospective study was conducted using anonymized patient data, and the requirement for informed consent was waived by the ethics committee. The study was conducted in accordance with the principles of the Declaration of Helsinki.

## Results

In this study, 98 patients diagnosed with Hodgkin lymphoma at the Pediatric Oncology Clinic of Samsun Ondokuz Mayıs University between 2005 and 2025 were evaluated. Sixteen patients were excluded because of insufficient data, and analyses were conducted on the remaining 82 patients. Among these, 49 (59.8%) were male and 33 (40.2%) were female. The mean age at diagnosis was 145.8 ± 54.94 months, with a median of 167.5 months (range: 29–216). According to the Ann Arbor staging system, 4 patients (4.9%) were classified as stage 1, 31 (37.8%) as stage 2, 23 (28%) as stage 3, and 24 (29.3%) as stage 4. B symptoms were present in 17 patients (20.7%) and absent in 65 (79.3%).

The initial site of disease involvement was cervical, axillary, or supraclavicular lymphadenopathy in 63 patients (76.8%). Mediastinal involvement was observed in 13 patients (15.9%), whereas abdominal involvement was noted in 4 patients (4.9%). Bulky disease was present in 14 patients (17.1%). An evaluation of the histological subtypes of Hodgkin lymphoma revealed that 58 patients (70.7%) had nodular sclerosis, 16 (19.5%) had mixed cellularity, 7 (8.5%) had predominant nodular lymphocytes, and 1 patient (1.2%) had the interfollicular subtype.

Laboratory findings revealed leukopenia (WBC < 4000/mm³) in 5 patients (6.1%) and leukocytosis (WBC > 12000/mm³) in 29 patients (35.4%). Hemoglobin levels were below 10 g/dL in 18 patients (22.0%), and platelet count was below 150,000/mm³ in 1 patient (1.2%).

Chemotherapy regimens including OPPA, OEPA, COPP, and ABVD were administered according to risk stratification and drug availability. Autologous stem cell transplantation was performed in 9 patients (11.0%), and radiotherapy was given to 46 patients (56.1%). In nine patients (11.0%), the early response after the first two chemotherapy cycles was inadequate on the basis of interim PET evaluation. The mean follow-up duration was 72.73 ± 49.53 months, with a median of 63.5 months (range: 7–232). During follow-up, relapse occurred in 13 patients (15.9%), refractory disease developed in 3 patients (3.7%), and 4 patients (4.9%) died. The 2-year overall survival (OS) rate was 96%, event-free survival (EFS) was 90%, the 5-year OS was 96%, and EFS was 85%.

The NLR at diagnosis was 3.76 ± 2.77, with a median value of 3.12 (range: 0.76–13.3). Poor clinical outcomes, defined as relapse, refractory disease, or death, were identified in 19 patients (23.2%). ROC curve analysis was performed to evaluate the discriminative ability of the NLR in predicting adverse outcomes. The area under the ROC curve (AUC) was 0.517 (95% CI: 0.373–0.661; *p* = 0.822), indicating poor diagnostic performance. Given the poor discriminative power of the NLR in the ROC analysis, the NLR was retained for further analysis in two distinct formats to ensure robust evaluation and comparison with the literature. For categorical assessments, the median value of 3.12 was selected as the cutoff to ensure an objective and balanced patient distribution, which is consistent with the cutoff ranges reported in previous studies assessing NLR in both adult and pediatric Hodgkin lymphoma patients [2, 10, 15]. Conversely, in the multivariable logistic regression model, the NLR was analyzed as a continuous variable to utilize its full numerical variance and avoid loss of statistical power.

When patients were stratified into two groups on the basis of the median NLR (3.12), no statistically significant association was found between the NLR and the occurrence of adverse outcomes (*p* = 0.500). However, several clinical and laboratory parameters demonstrated statistically significant differences between the groups. Patients with high NLRs (> 3.12) had significantly greater diagnostic ages, white blood cell counts, platelet counts, sedimentation rates, and CRP levels, but lower hemoglobin, and albumin levels (all *p* < 0.05) (Table 1).

**Table 1:** Demographic, clinical, and laboratory characteristics of pediatric patients with Hodgkin lymphoma stratified by diagnostic neutrophil-to-lymphocyte ratio (NLR)

		NLR ≤ 3.12 (*n* = 41)		NLR > 3.12 (*n* = 41)		Patients (*n* = 82)		*p*	
		Mean ± SD / *n*-%	Median (Min-Max)	Mean ± SD / *n*-%	Median (Min-Max)	Mean ± SD / *n*-%	Median (Min-Max)		
Age at diagnosis (months)		121.95 ± 9.21	125 (29–212)	169.66 ± 5.98	176 (55–216)	145.8 ± 54.94	167.5 (29–216)	***< 0.001***	m
Sex	Male	32 (65.3%)		17 (34.7%)		49 (59.8%)		***0.001***	x²
	Female	9 (27.3%)		24 (72.7%)		33 (40.2%)			
Stage	I	4 (100%)		0 ( 0%)		4 (4.9%)		***0.031***	x²
	II	17 (54.8%)		14 (45.2%)		31 (37.8%)			
	III	13 (56.5%)		10 (43.5%)		23 (28%)			
	IV	7 (29.2%)		17 (70.8%)		24 (29.3%)			
B symptoms	Present	7 (41.2%)		10 (52.8%)		17 (20.7%)		0.293	x²
	Absent	34 (52.3%)		31 (47.7%)		65 (79.3%)			
White Blood Cell Count (/mm3)		7,500 ± 477	7,250 (2,690 − 17,450)	13,193 ± 710	13,290 (4,580 − 22,910)	10,346 ± 4,798	9,380 (2,690 − 22,910)	***< 0.001***	m
Hemoglobin (g/dL)		12.12 ± 0.27	12.3 (6.6–15.8)	10.64 ± 0.27	11.1 (6.4–14.1)	11.38 ± 1.89	11.6 (6.4–15.8)	***< 0.001***	m
Platelet Count (/uL)		320.85 ± 15.34	310 (168–589)	434.44 ± 22.11	439 (139–723)	377.65 ± 133.91	337 (139–723)	***< 0.001***	m
Neutrophil Count (/mm3)		4,029 ± 326	3,640 (1,070 − 11,440)	10,142 ± 582	9,940 (3,270 − 19,440)	7,986 ± 4,300	5,755 (1,070 − 19,440)	***< 0.001***	m
Lymphocyte Count (/mm3)		2,530 ± 164.84	2,280 (1,050 − 5,400)	1,921 ± 127	1,930 (430–4,200)	2,225 ± 987	2,070 (430–5,400)	***0.013***	m
Monocyte Count (/mm3)		594.15 ± 46.35	510 (10 − 1,260)	812.44 ± 48.96	770 (210–1,700)	703.29 ± 322.65	640 (10 − 1,700)	***0.002***	t
Neutrophil-to-Lymphocyte Ratio (NLR)		1.66 ± 0.1	1.62 (0.76–3.1)	5.86 ± 0.38	5.01 (3.15–13.3)	3.76 ± 2.77	3.12 (0.76–13.3)		
Sedimentation (mm/hr)		31.93 ± 4.17	24 (2–110)	65.88 ± 5.52	60 (7–140)	48.9 ± 35.51	42.5 (2–140)	***< 0.001***	m
Albumin (g/dL)		4.39 ± 0.06	4.51 (3.3–5.14)	4.02 ± 0.08	4.2 (2.3–5.1)	4.20 ± 0.52	4.25 (2.3–5.1)	***0.001***	m
CRP (mg/dL)		20.93 ± 5.53	3 (1–131)	72.41 ± 9.1	76 (1–190)	46.67 ± 54.46	20 (1–190)	***< 0.001***	m
LDH (U/L)		319.22 ± 14.8	293 (187–645)	317.88 ± 24.4	271 (159–830)	318.55 ± 128.43	282.5 (159–830)	0.189	m
Relapsed/Refractory or Deceased	Yes	9 (47.4%)		10 (52.6%)		19 (23.2%)		0.500	x²
	No	32 (50.8%)		31 (49.2%)		63 (76.8%)			

*NLR* Neutrophil-to-Lymphocyte Ratio; x² = Chi-square; u= Mann–Whitney U; t= Student’s t-test; *p*-values were obtained using the x² test for categorical variables and the Mann–Whitney U or Student’s t-test for continuous variables, as appropriate; Bold values indicate statistically significant results (*p* < 0.05)

**Fig. 1:**
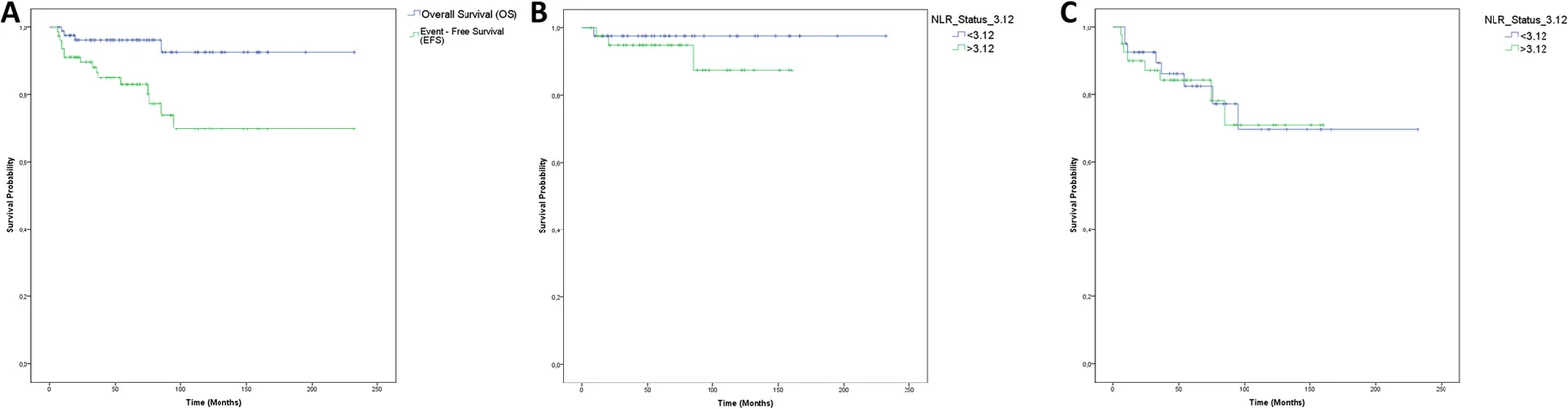
Kaplan–Meier curves for overall survival and event-free survival according to the NLR. **A** Overall survival and event-free survival for the entire cohort. **B** Overall survival according to the NLR (log-rank p = 0.315). **C** Event-free survival according to the NLR (log-rank p = 0.870)

Kaplan–Meier survival analysis with the log-rank test revealed no statistically significant difference between the low and high NLR groups in terms of OS (*p* = 0.315) or EFS (*p* = 0.870) (Fig. 1).

Univariable logistic regression analysis was performed to evaluate the relationships between demographic, clinical, and laboratory variables and adverse clinical outcomes. Among the examined parameters, bulky disease and two-cycle chemotherapy response were significantly associated with poor prognosis. Patients with inadequate early response after two chemotherapy cycles had a markedly greater risk of poor outcomes (OR = 0.056; 95% CI: 0.010–0.304; *p* = 0.001), whereas bulky disease showed a borderline association (OR = 3.173; 95% CI: 0.938–10.736; *p* = 0.063). In contrast, the NLR was not significantly associated with adverse outcomes (OR = 0.97; 95% CI: 0.80–1.18; *p* = 0.754) (Table 2).

**Table 2:** Logistic regression analysis of factors associated with adverse clinical outcomes

	OR	Univariate Model	*p*	OR	Multivariate Model	*p*
		95% Confidence Interval (CI)			95% Confidence Interval (CI)	
Age	0.997	0.987–1.006	0.461			
Sex	1.462	0.520–4.114	0.471			
Stage	1.456	0.816–2.598	0.203	1.522	0.719–3.222	0.273
B Symptoms	0.376	0.078–1.820	0.224	0.365	0.063–2.123	0.262
White Blood Cell	0.908	0.804–1.026	0.121			
Hemoglobin	0.973	0.742–1.276	0.843			
Platelet	0.998	0.993–1.002	0.249			
Neutrophil	0.908	0.793–1.040	0.164			
Lymphocyte	0.713	0.399–1.273	0.252			
Monocyte	0.618	0.119–3.208	0.567			
Erythrocyte Sedimentation Rate (ESR)	1.000	0.986–1.015	0.954	0.996	0.974–1.019	0.718
C-Reactive Protein (CRP)	1.003	0.994–1.012	0.496			
Lactate Dehydrogenase (LDH)	1.000	0.996–1.004	0.927			
Albumin	0.561	0.217–1.454	0.234	0.455	0.105–1.976	0.294
Neutrophil-to-Lymphocyte Ratio (NLR)	0.970	0.800–1.175	0.754	0.891	0.681–1.167	0.403
Bulky Disease	3.173	0.938–10.736	0.063	4.405	0.946–20.516	***0.059***
Two-cycle response	0.056	0.010–0.304	***0.001***	0.038	0.005–0.293	***0.002***

To further assess the potential independent prognostic role of clinical and laboratory variables while minimizing overfitting risk, a parsimonious multivariable logistic regression model was constructed, including stage, B symptoms, albumin, ESR, NLR, bulky disease, and two-cycle chemotherapy response. In this adjusted model, an inadequate early response after two chemotherapy cycles remained a statistically significant independent predictor of adverse outcomes (OR = 0.038; 95% CI: 0.005–0.293; *p* = 0.002), whereas bulky disease showed a borderline association (OR = 4.405; 95% CI: 0.946–20.516; *p* = 0.059). None of the other parameters, including the NLR, retained statistical significance (OR = 0.891; 95% CI: 0.681–1.167; *p* = 0.403).

## Discussion

The core finding of this study is the lack of a significant association between the pretreatment NLR and adverse clinical outcomes (relapse, refractory disease, or mortality) in pediatric HL patients. Despite the NLR being a simple, universally available, and inexpensive biomarker widely accepted as a reflection of systemic inflammation, both univariable and multivariable analyses indicated that the NLR was not an independent predictor of adverse outcomes [4, 5, 7, 16]. Similarly, conventional parameters that are often studied, such as stage, albumin, and erythrocyte sedimentation rate, also failed to predict adverse outcomes in this cohort [1]. These findings suggest that in our cohort, baseline hematologic and biochemical markers failed to provide prognostic stratification despite their established use in risk assessment models. Given that our institution serves as a tertiary referral center, the proportion of advanced-stage (stage IV) patients, bulky disease patients, and autologous stem cell transplantation patients was relatively high, which may have influenced the overall prognostic distribution of our population.

This finding stands in stark contrast to those of numerous adult studies. In adult classical HL cohorts, a high NLR is repeatedly identified as an independent factor associated with poor survival [3, 7, 9, 10]. An elevated NLR is correlated with unfavorable features such as advanced stage disease, B symptoms, shortened OS and progression-free survival (PFS) [3, 7, 9, 10]. The prognostic significance of the NLR has been robustly confirmed across many adult solid tumors [4, 5, 17]. Unlike most adult studies, however, our analysis, which is consistent with similar findings reported by major pediatric groups, suggested that baseline inflammatory indices were insufficient for distinguishing adverse clinical outcomes in this cohort [1, 2]. In our series, even though patients with high NLRs presented high levels of inflammatory markers such as ESR and CRP, these elevations did not translate into poor survival outcomes. Interestingly, unlike traditional risk factors such as stage, B symptoms, and inflammatory markers, which were not significant in our analysis, bulky disease and early chemotherapy response after two cycles emerged as meaningful predictors of outcome. Even in adult series employing an interim PET-2–based strategy, the independent contribution of the NLR is largely superseded by interim PET-2 response, retaining only limited, advanced-stage–restricted prognostic value [18]. This finding suggests that in pediatric HL, dynamic tumor characteristics (such as initial tumor burden and early metabolic response) may better reflect disease biology and treatment sensitivity than static baseline laboratory parameters.

The divergence observed between pediatric and adult cohorts stems from fundamental biological and clinical differences. Pediatric HL generally has excellent outcomes, with OS rates frequently exceeding 90% [1]. Consequently, the event definition was set as relapse/refractory disease or death due to the high overall survival observed in Hodgkin lymphoma patients. Despite this meticulous definition, few events (23.2%) were observed, and this excellent outcome may have statistically complicated the robust identification of prognostic factors. This high cure rate inherently diminishes the discriminatory power of most baseline biomarkers [1, 2]. Moreover, the higher prevalence of bulky and advanced-stage disease in our referral-based cohort could have led to a relative homogenization of clinical risk, limiting the statistical distinction between traditional prognostic factors.

Furthermore, the immune systems of pediatric and adult patients may not be at the same maturity level, and Hodgkin lymphoma may arise through biologically distinct mechanisms in the two groups. The tumor microenvironment in children may be less immunosuppressive, facilitating a more effective antitumor T-cell response [2]. Additionally, NLRs levels in adults are frequently confounded by comorbidities and chronic inflammatory stimuli that are typically absent in children (such as chronic infection or cardiovascular disease) [4, 16, 19, 20]. Therefore, the NLR may not accurately reflect the balance between tumor-induced inflammation and immune surveillance in pediatric patients [2]. These results strongly suggest that the inflammatory response in pediatric patients is biologically different from that in adults. This biological rationale aligns with our findings, where baseline inflammatory activation reflected by the NLR and CRP did not correlate with disease aggressiveness or survival.

From a methodological perspective, the robust finding that the NLR is not an important independent marker is strengthened by analyzing the NLR via both continuous and categorical approaches, which reduces the possibility of threshold bias. This is consistent with the AIEOP LH-2004 pediatric cohort, in which the NLR (ANC/ALC ratio) showed prognostic relevance in univariable analysis but was not retained in the final multivariable model, unlike stage IV disease, ferritin, eosinophils, and platelets [1]. Taken together, our results indicate that systemic inflammatory markers such as the NLR, although correlated with inflammatory parameters, do not provide meaningful prognostic information in pediatric patients with HL.

The limited prognostic utility of the NLR observed in pediatric HL patients should be interpreted within the broader context of pediatric malignancies, where the results vary widely. While its prognostic value appears limited in HL, inflammation-based ratios such as the NLR, PLR (platelet-to-lymphocyte ratio), and MLR (monocyte-to-lymphocyte ratio) may still have diagnostic or disease-monitoring relevance, particularly in distinguishing malignant lymphadenopathy (including HL) from benign reactive conditions in children [11, 12, 21]. Moreover, high NLR values have been identified as independent predictors of poor OS and PFS in aggressive pediatric solid tumors, such as osteosarcoma, Ewing sarcoma, and sinonasal rhabdomyosarcoma, highlighting that the prognostic role of inflammation is highly tumor type-specific [2, 20, 22].

This study has several limitations, including its retrospective design, single-center nature, relatively small sample size, and limited number of adverse events, which may have reduced the statistical power needed to detect subtle effects. We also did not assess dynamic changes in the NLR during therapy, which might provide additional prognostic insights, as monitoring changes in the NLR over time has been shown to be predictive of therapeutic response in other cancers [16, 23]. Nevertheless, the study’s long follow-up period and comprehensive inclusion of clinical and laboratory variables strengthen the validity of our findings.

In conclusion, the NLR at diagnosis did not show prognostic value in this cohort. The observation that both inflammatory markers and traditional clinical factors have limited prognostic discrimination in the current risk-adapted treatment era suggests that caution should be exercised when directly applying inflammatory markers (e.g., the NLR) derived from adult cohorts to childhood malignancies, and the development of immune parameters specific to the pediatric age group should be targeted. Future multicenter studies with larger cohorts and prospective designs are warranted to clarify the clinical utility of systemic inflammation markers in pediatric HL patients.

## Data Availability

The datasets generated and/or analyzed during the current study are not publicly available due to institutional privacy and data protection policies but are available from the corresponding author on reasonable request.

## Abbreviations

NLR: Neutrophil-to-Lymphocyte Ratio
HL: Hodgkin Lymphoma
ESR: Erythrocyte Sedimentation Rate
ROC: Receiver Operating Characteristic
AUC: Area Under Curve
LDH: Lactate Dehydrogenase
CRP: C-reactive Protein
PET: Positron Emission Tomography
WBC: White Blood Cell
OPPA: Oncovin, Procarbazine, Prednisone, Adriamycin
OEPA: Oncovin, Etoposide, Prednisone, Adriamycin
COPP: Cyclophosphamide, Oncovin, Procarbazine, Prednisone
ABVD: Adriamycin, Bleomycin, Vinblastine, Dacarbazine
OS: Overall Survival
EFS: Event-Free Survival
PFS: Progression-Free Survival
OR: Odds Ratio
PLR: Platelet-to-Lymphocyte Ratio
LMR: Lymphocyte-to-Monocyte Ratio
CI: Confidence Interval
